# Supplementary material for: Splice-Junction-Based Mapping of Alternative Isoforms in the Human Proteome
Source: Cell Rep. Author manuscript; Available in PMC 2020 Jan 15. (PMC6961840; doi:10.1016/j.celrep.2019.11.026)
Supplement: 2 [file NIHMS1546469-supplement-2.zip › DF1/PXD006675/LeftAtrium/LeftAtrium_38_ANKRD1_DELESTAIHWASR.pdf]

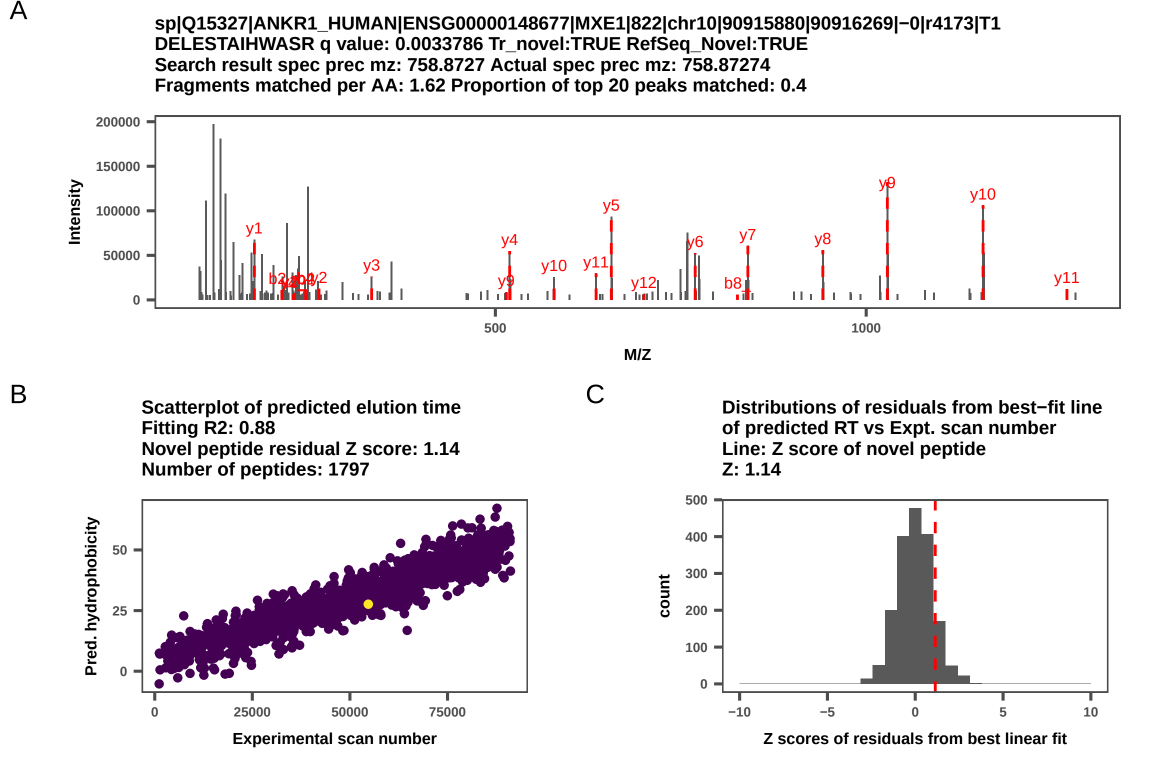
A
sp|Q15327|ANKR1_HUMAN|ENSG00000148677|MXE1|822|chr10|90915880|90916269|−0|r4173|T1
DELESTAIHWASR q value: 0.0033786 Tr_novel:TRUE RefSeq_Novel:TRUE
Search result spec prec mz: 758.8727 Actual spec prec mz: 758.87274
Fragments matched per AA: 1.62 Proportion of top 20 peaks matched: 0.4
Intensity
M/Z
B
Scatterplot of predicted elution time
Fitting R2: 0.88
Novel peptide residual Z score: 1.14
Number of peptides: 1797
Pred. hydrophobicity
Experimental scan number
C
Distributions of residuals from best−fit line
of predicted RT vs Expt. scan number
Line: Z score of novel peptide
Z: 1.14
count
Z scores of residuals from best linear fit
